# Alteration in the *Culex pipiens* transcriptome reveals diverse mechanisms of the mosquito immune system implicated upon Rift Valley fever phlebovirus exposure

**DOI:** 10.1371/journal.pntd.0008870

**Published:** 2020-12-10

**Authors:** Ana I. Núñez, Anna Esteve-Codina, Jèssica Gómez-Garrido, Marco Brustolin, Sandra Talavera, Miguel Berdugo, Marc Dabad, Tyler Alioto, Albert Bensaid, Núria Busquets

**Affiliations:** 1 IRTA, Centre de Recerca en Sanitat Animal (CReSA, IRTA-UAB), Campus de la Universitat Autònoma de Barcelona, Bellaterra, Spain; 2 CNAG-CRG, Centre for Genomic Regulation (CRG), Barcelona Institute of Science and Technology (BIST), Barcelona, Catalonia, Spain; 3 Department of Entomology, The Pennsylvania State University, University Park, Pennsylvania, United States of America; 4 Instituto de Biología Evolutiva, Universitat Pompeu i Fabra-CSIC, Dr. Aigüader 88, Barcelona, Spain; 5 Universitat Pompeu i Fabra (UPF), Barcelona, Catalonia, Spain; The Pennsylvania State University, UNITED STATES

## Abstract

Rift Valley fever phlebovirus (RVFV) causes an emerging zoonotic disease and is mainly transmitted by *Culex* and *Aedes* mosquitoes. While *Aedes aegypti*-dengue virus (DENV) is the most studied model, less is known about the genes involved in infection-responses in other mosquito-arboviruses pairing. The main objective was to investigate the molecular responses of *Cx*. *pipiens* to RVFV exposure focusing mainly on genes implicated in innate immune responses. Mosquitoes were fed with blood spiked with RVFV. The fully-engorged females were pooled at 3 different time points: 2 hours post-exposure (hpe), 3- and 14-days post-exposure (dpe). Pools of mosquitoes fed with non-infected blood were also collected for comparisons. Total RNA from each mosquito pool was subjected to RNA-seq analysis and a *de novo* transcriptome was constructed. A total of 451 differentially expressed genes (DEG) were identified. Most of the transcriptomic alterations were found at an early infection stage after RVFV exposure. Forty-eight DEG related to immune infection-response were characterized. Most of them were related with the RNAi system, Toll and IMD pathways, ubiquitination pathway and apoptosis. Our findings provide for the first time a comprehensive view on *Cx*. *pipiens*-RVFV interactions at the molecular level. The early depletion of RNAi pathway genes at the onset of the RVFV infection would allow viral replication in mosquitoes. While genes from the Toll and IMD immune pathways were altered in response to RVFV none of the DEG were related to the JAK/STAT pathway. The fact that most of the DEG involved in the Ubiquitin-proteasome pathway (UPP) or apoptosis were found at an early stage of infection would suggest that apoptosis plays a regulatory role in infected *Cx*. *pipiens* midguts. This study provides a number of target genes that could be used to identify new molecular targets for vector control.

## Author summary

Rift valley fever (RVF) is an emerging zoonotic disease and it is caused by RVFV. This virus is commonly transmitted in endemic areas between wild ruminants and mosquitoes, mainly by mosquitoes of *Culex* and *Aedes* genus. Starting from the year 2000, several outbreaks have been reported outside Sub Saharan Africa, in countries facing the Mediterranean Sea (Egypt), or Yemen and Saudi Arabia. Available vaccines for ruminants present limited efficacy or residual pathogenic effects. Consequently, new strategies are urgently required to limit the expansion of this zoonotic virus. The main objective of this work is to investigate transcriptional alterations of *Cx*. *pipiens* to RVFV focusing mainly on genes implicated in conventional innate immunity pathways, RNAi mechanisms and the apoptotic process in order to evaluate the involvement of these genes in viral infection. The immune altered genes here described could be potential targets to control RVFV infection in mosquitoes. Some of the genes related to the immune defense response were previously described in others mosquito-arbovirus models, as well as in *Drosophila* and human. To our knowledge, this study highlights for the first time the *Cx*. *pipiens*-RVFV interactions in terms of defense infection-response and provides information for developing in the future new approaches to prevent and control the expansion of the virus.

## Introduction

Rift Valley fever (RVF) is an emerging arthropod-borne zoonotic disease caused by an enveloped RNA segmented phlebovirus, RVFV. Beside wild ruminants, RVFV affects mainly domestic ruminants and humans [1]. It was first described in 1930 in the Rift Valley (Kenya), after observing high abortion and mortality rates in sheep near Lake Naivasha [2]. In 1948, mosquitoes of the *Aedes* genus were identified as RVFV vectors in Uganda [3]. Since then, the virus has been isolated from at least 53 mosquito species covering 8 genera, mainly *Aedes* and *Culex* genus, and belonging to the Culicidae family [4]. After successive outbreaks in eastern and southern Africa, RVFV was detected in Egypt in 1977, causing a major human outbreak involving *Cx*. *pipiens* species as the main potential vector [4]. RVFV was identified for the first-time outside Africa in 2000, in Saudi Arabia and Yemen. During this outbreak, the impact on the economy and public health was very high, reaching a total of 882 confirmed cases with 124 deaths [5]. The spread of RVFV outside the African continent and its presence in Egypt, which faces the Mediterranean Sea, highlighted the possibility of RVFV introduction in Europe [6]. Indeed, European autochthonous vectors, such as *Cx*. *pipiens* or the invasive Asian tiger mosquito, *Aedes albopictus* have been proven to be competent vectors under laboratory conditions [7,8]. Currently, RVFV is circulating in several African countries causing sporadic outbreaks [9] and according to WHO, it is a prioritized emerging infectious disease.

Mosquitoes within the *Cx*. *pipiens* complex are known to be competent vectors for RVFV [8,10,11]. The capacity of the mosquitoes to acquire, replicate and transmit an arbovirus, known as vector competence, depends on: i) extrinsic factors, such as environmental conditions [12] or genetic variations of the pathogens [7], and ii) intrinsic factors, such as the genetics of vector populations [13], and the immune system of each mosquito strain [14]. Multiple immune effector mechanisms in insect are implicated in the defense against microorganisms: apoptosis, encapsulation, melanization, phagocytosis of the pathogens and the production of antimicrobial peptides (AMP) [15–18]. Immune effectors are triggered mainly by three innate immunity pathways: Toll, Immune deficiency (IMD) and Janus kinase/signal transducer and activator of transcription (JAK/STAT). All of them are involved in the defense against arbovirus infection [16]. The Toll pathway is activated after the interaction between the antigen and the Toll-like receptors. This eventually induces the expression of antiviral effectors [16]. Several functional assays have shown that the Toll signaling pathway plays a key role for DENV control in infected *Ae*. *aegypti* mosquitoes [16,19,20]. Moreover, activation of the Toll pathway in the midgut of *Ae*. *aegypti* infected with the Sindbis virus (SINV) and Zika virus (ZIKV) has also been suggested [21,22]. Additionally, there is mounting evidence for the antiviral function of the IMD signaling pathway in mosquitoes [14]. The IMD pathway is triggered upon virus infection recognition by the adaptor IMP protein, resulting in the transcription of IMD effector genes [16]. Upon DENV infection, the salivary glands of *Ae*. *aegypti* secrete an IMD dependent antimicrobial peptide (AMP) with antiviral activity against DENV and Chikungunya virus (CHIKV) [23]. The invertebrate JAK/STAT pathway is similar to the mammalian interferon response. The JAK/STAT pathway is also involved in *Ae*. *aegypti* defense responses against DENV and ZIKV infection [14,22]. Elements of different immune pathways may act synergistically and interact with components of apoptosis and other metabolic pathways. Apoptosis is a defense mechanism that insects use against viral infections by killing infected or neighboring cells. Programmed cell death is involved, at the midgut level, in the control of DENV and West Nile virus (WNV) infections in *Ae*. *aegypti* [24] and *Cx*. *pipiens* [25] respectively. On the other hand, Patel *et al*. [26], reported that the phosphatidylinositol 3-kinase (PI3K)-Akt-TOR signaling pathway promotes viral replication in insect cells during SINV infection and causes inhibition of apoptosis. Although the above-mentioned transduction pathways are relevant for controlling arbovirus infections, the RNA interference (RNAi) pathway is considered to be the major innate immune response against viral infections [24]. The small interfering RNA (siRNA) pathway is the best known regarding its role in restricting arbovirus infections (i.e. DENV, SINV and CHIKV) in *Ae*. *aegypti* [27–29], but it can also generate virus diversification during WNV infection in *Cx*. *quinquefasciatus* [30]. Additionally, the P-element-induced Wimpy testes gene (PIWI)-interacting RNA (piRNA) pathway, which is involved in maintaining genomic integrity, has also been suggested to be implicated in antiviral defense [31].

The importance of the mosquito innate immune system against different flaviviruses, specifically against DENV has been studied in detail. However, less is known on the interaction of bunyaviruses such as RVFV with the mosquito immune system. In the present study, the effect of the RVFV in exposed *Cx*. *pipiens* has been studied using RNA-seq analysis. *Culex pipiens* was chosen for the present study because of its wide geographical distribution and its capacity to transmit RVFV [8]. Analysis were performed at 2 hours post-exposure (hpe), 3- and 14-days post-exposure (dpe) to observe changes in gene expression induced by signaling pathways just after viral exposure (2 hpe) and at both early (3 dpe) and late (14 dpe) stages of viral infection. Differentially expressed genes related to the mosquito’s immune system response have been identified after RVFV exposure. This is the first study that analyzes the *Cx*. *pipiens* transcriptome upon RVFV exposure in order to better understand interactions between *Cx*. *pipiens* mosquitoes and RVFV and potential mechanisms involved in the control of RVFV infection.

## Materials and methods

### Mosquitoes rearing

*Culex pipiens* hybrid form mosquitoes used for RVFV infection were reared from a laboratory colony originally collected in Gavà in 2012 (Spain). The molecular genetic characterization of the mosquitoes was performed in a previous study [8]. Mosquitoes(generation 43) were reared under environmental conditions simulating those found around their natural breeding sites, at 26°C during the day and 22°C at night at 80% of relative humidity (RH). The photoperiod consisted in 14 h: 10 h (light: dark) with two crepuscular cycles of 30 min to simulate dawn and dusk [8]. Mosquitoes were fed using 10% sucrose solution.

### Virus production and titration

A virulent RVFV strain (RVF 56/74), kindly provided by Dr. Alejandro Brun (CISA-INIA) and originally isolated from cattle in 1974 [32], was used for mosquito experimental infection. RVF 56/74 was propagated in C6/36 cells and titrated in Vero cells as previously described [8].

### Experimental RVFV infection in *Culex pipiens* mosquitoes

After adult mosquito emergence, mosquitoes were fed *ad libitum* on a 10% sucrose solution until 30 hours before blood feeding, when the sucrose solution was removed. Mosquitoes were kept in 0.5 L volume plastic cages with mesh screening during 48 hours before mosquitoes were feed with blood. Three hundred adult females of 7-10- days-old were used for the present study. One hundred fifty adult females were exposed to infectious blood and 150 females were exposed to non-infectious blood. The heparinized bovine blood mixed with adenosine 5’-triphosphate (ATP) (5x10^-3^ M) (Sigma-Aldrich, St. Louis, MO) was extracted from bovine (medial caudal vein) and was provided to mosquitoes using the Hemotek artificial system (Discovery Workshop, Accrington, UK) at 37.5°C ± 0.5. Aliquots of RVFV-spiked blood were collected after feeding and used to calculate the viral titer using the TCID_50_ assay in Vero cell (RVFV titer blood = 7.46 log_10_ TCID_50_/mL).

The fully engorged females were anaesthetized using CO_2_, selected and maintained in individual cardboard cages (Watkins & Doncaster, Leominster, UK) sealed with a net on top and stored inside a climatic chamber under rearing environmental conditions. Throughout the experiment, the mosquitoes were maintained with permanent access to 10% sucrose solution. Twelve fully-engorged females from each group (infectious and non-infectious blood) were removed at 2 hpe, and at 3 and 14 dpe. These females were anaesthetized using CO_2_ and stored at -80°C until use. The experimental infection was performed in Biosafety Level 3 facilities at the *Centre de Recerca en Sanitat Animal* (IRTA-CReSA).

### Total RNA extraction and RVFV detection

Fully engorged females were pooled in groups of four. Three pools were used for each time point (2 hpe, 3 dpe and 14 dpe) and condition: exposed to infectious (hereafter referred to as RVFV) and non-infectious blood (hereafter referred to as control). In total, 18 pools of four females were obtained. The entire mosquitoes were homogenized with glass beads for 2 min at 30 Hz using TissueLyser II (Qiagen GmbH, Hilden, Germany) and total RNA was extracted from each pool using RNeasy Mini Kit (250) (Qiagen GmbH, Hilden, Germany). Total RNA was eluted using 50 μL of RNase-free water.

RVFV RNA was detected by reverse transcription quantitative PCR (RT-qPCR) using primers defined previously [33] and AgPath-ID One-Step RT-PCR Reagents (Applied Biosystems, Inc., Foster City, CA, U.S.A.) following the amplification protocol previously described [8]. The limit of sensitivity was 0.09 TCID_50_ per reaction, which corresponded to a Ct value of 39.19.

### RNA library preparation and sequencing

Total RNA from *Cx*. *pipiens* samples was assayed for quantity and quality using Qubit RNA HS Assay (Life Technologies) and RNA 6000 Nano Assay on a Bioanalyzer 2100 (Agilent).

The RNASeq libraries were prepared from total RNA using the TruSeq RNA Sample Prep Kit v2 (Illumina Inc., Rev. E, October 2013). Briefly, after poly-A based mRNA enrichment with oligo-dT magnetic beads of at least 0.52 μg of total RNA as input material, the mRNA was fragmented to 80–250 nt, with the major peak at 130 nt. The second strand cDNA synthesis was performed in the presence of dUTP instead of dTTP, to achieve the strand specificity. The blunt-ended double stranded cDNA was 3´adenylated and Illumina indexed adapters were ligated. The ligation product was enriched with 15 PCR cycles and the final library was validated on an Agilent 2100 Bioanalyzer with the DNA 7500 assay.

The libraries were sequenced on HiSeq2000 (Illumina, Inc) in paired-end mode with a read length of 2x76 bp using the TruSeq SBS Kit v4 (Illumina). Image analysis, base calling and quality scoring of the run were processed using the manufacturer’s software Real Time Analysis (RTA 1.18.66.3) and followed by generation of FASTQ sequence files by CASAVA.

### *De novo* transcriptome assembly and annotation

Transcriptome assemblies were performed for each time point (2 hpe, 3 dpe or 14 dpe) with Trinity v2.2.0 [34], with read trimming and normalization options activated. Afterwards, the Rapclust [35] approach was followed on each of the Trinity assemblies in order to reduce redundancy before merging the three resulting transcriptomes together with Rapclust. In this process, a pseudoalignment is first performed with Sailfish v0.10.0 [36] and then Rapclust V0.1 is used to cluster the assembled sequences into contained isoforms, in order to reduce redundancy and to cluster together all the isoforms that are likely to belong to the same gene.

After obtaining the reference transcriptome, Open Reading Frames were annotated in the assembled transcripts with Transdecoder [37]. Next, we used Trinotate v3.0.1 [38] to functionally annotate the protein-coding transcripts. First, BLAST [39] searches were performed against the Swiss-prot database (last accessed Nov 2016). Moreover, the program HMMER and Signalp [40] were also used to detect protein domains in the predicted ORFs. Finally, the outputs of all these programs were combined into an SQL database by using Trinotate.

### Transcript quantification and differential expression analysis

RNA-seq reads were mapped against the *de novo* assembled transcriptome with the program rsem-extract-reference from RSEM [41] using STAR [42] indices and the option -transcript-to-gene-map to map transcripts (contigs) to genes (clusters). Genes were quantified with RSEM using our custom gene annotation. As exploratory analysis, a principal component analysis (PCA) with the top 500 most variable genes was performed with the ‘prcomp’ function and ‘ggplot2’ (S1 Fig). Differential expression analysis was performed with the DESeq2 default normalization and multiple test correction provided by the package. [43]. Genes with adjusted P-value <0.05 were considered significant. Gene ontology enrichment of the differentially expressed genes was performed with TopGO [44] with a cut-off of ‘classicFisher’ P-value <0.05. CirGO [45] was used to visualize the GO enrichment results. Gene ontology terms based on statistical significance were summarized with a semantic similarity algorithm based on Revigo [46] and grouped by hierarchical clustering.

**Fig 1:**
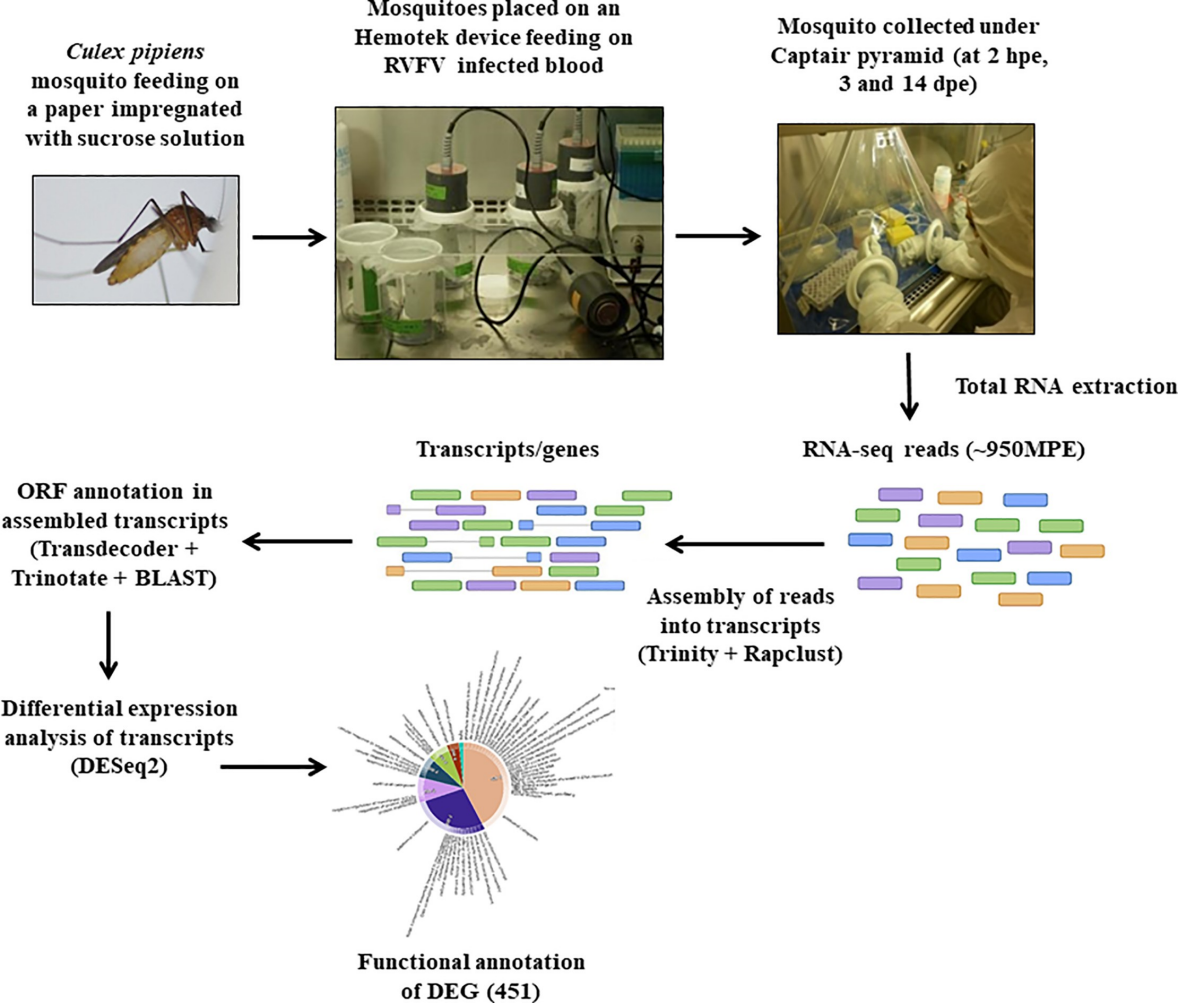
The workflow of the experimental infection in *Cx*. *pipiens* mosquitoes exposed to RVFV and transcriptome analysis.

The workflow of the experimental infection in *Cx*. *pipiens* mosquitoes exposed to RVFV and transcriptome analysis is shown in the Fig 1.

### Identification of differentially expressed genes related to immunity

Differentially expressed genes (hereafter DEG) coding for proteins involved in mosquito innate immunity were identified by screening two databases. First, a local database of *Cx*. *quinquefasciatus* proteins involved in immunity was created by retrieving the *ad hoc* entries from the ImmuneDB data base [47] for mosquito species. This database is available under request. BLASTX BioEdit [39,48] was used to compare *Cx*. *quinquefasciatus* immunity-related proteins with our database of DEG of *Cx*. *pipiens*. Second, *Cx*. *pipiens* DEG related to antiviral/immunity responses were retrieved from our *Cx*. *pipiens de novo* assembled transcriptome annotated with the Gene Ontology database. All *Cx*. *pipiens* DEG identified by both methods were further curated by performing a BLASTX search of the nr protein database at the National Center for Biology Information (NCBI). The online NCBI BLASTX search also provided information on conserved protein domains by screening the CDD and pFAM databases for homologies [49] and UniProt was used to obtain information about the protein characteristics [50].

## Results

### RVFV infection in *Culex pipiens* mosquitoes

The three RVFV pools were positive for RVFV by RT-qPCR at the three selected time points (2 hpe, 3 dpe and 14 dpe) of study. As expected, all the control pools were negative by RT-qPCR (Table 1). At 2 hpe, the virus detected in the samples from the RVFV group was derived from the blood meal. These results demonstrated that each pool at 3 and 14 dpe had at least one RVFV infected mosquito female after exposure to infectious blood and that the infection was sustained for the entire 14 days of the experiment, although one mosquito pool (RVFV pool 3) at 14 dpe showed a lower level of infection.

**Table 1:** Viral load (Ct values) in mosquito pools tested by RT-qPCR specific to RVFV. Pools were composed of four mosquito females.

	Mosquito pool	2 hpe	3 dpe	14 dpe
Control	1	0	0	0
	2	0	0	0
	3	0	0	0
RVFV	1	23.13	27.09	24.3
	2	26.15	28.84	25.32
	3	21.9	25.47	33.88

### Transcriptome *de novo* assembly

To obtain a good reference transcriptome assembly to be used in further analysis we first assembled the reads that belonged to each time-point independently and then clustered all the transcriptomes. S1 Table shows the total number of raw reads, the uniquely mapped reads and multi-mapped reads among other informative QC metrics. A total of approximately 950 million reads were detected. The final assembly is composed of 355,773 assembled contigs with a contig N50 of 1716 nt. The transcripts clustered into 106,771 clusters, hereafter referred to and treated as “genes”, although the number is inflated due to the usual transcript fragmentation and duplication exhibited by transcriptome assemblies. Finally, 46,635 contigs are considered protein-coding. The gene completeness of the reference transcriptome was estimated with Benchmarking Universal Single-Copy Orthologs (BUSCO) v1.1b1 [51], using an arthropod specific dataset made of 2675 genes. According to BUSCO, our reference transcriptome has 92% of complete genes, 5.1% fragmented genes and only a 2.6% of missing genes. In total, 141,669 transcripts have been annotated as protein coding, of which 113,513 (80%) have been functionally annotated with protein descriptions and 97,283 (69%) with gene ontology (GO) terms.

### Comparisons of transcripts accumulation level in RVFV-exposed and non-exposed mosquitoes

**Fig 2:**
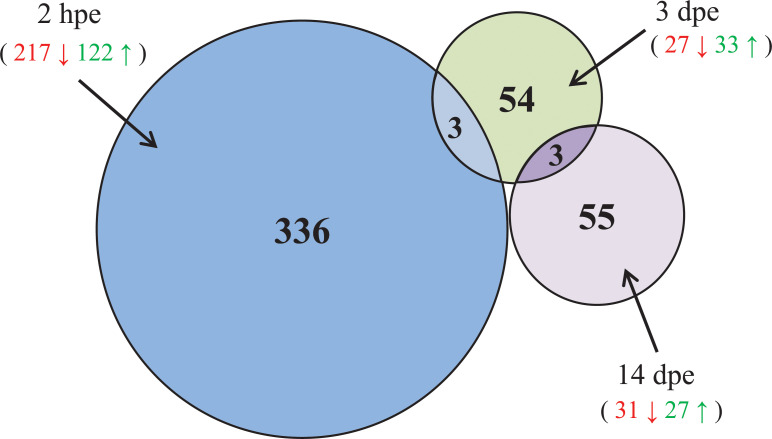
Venn diagram of DEG in RVFV infected *Cx*. *pipiens* mosquitoes. The Venn diagram represents the number of DEG in response to RVFV at different time-points (2 hpe, 3 dpe and 14 dpe). In red, the number of down-regulated genes, in green the number of up-regulated genes.

The abundance of genes in RVFV- exposed *versus* non- exposed *Cx*. *pipiens* mosquitoes were evaluated for each of the three different time points (2 hpe, 3 dpe and 14 dpe). Genes with adjusted p-value ≤ 0.05 and Fold Change (FC) ≥ + 1.5 or ≤ - 1.5 were considered significantly up-regulated or down-regulated. A total of 451 DEG were identified (Fig 2 and S2 Table). From the 451 DEG, 176 genes were up-regulated and 263 were down-regulated in RVFV exposed mosquitoes. Six DEG were up or down-regulated depending on the time-point. In RVFV exposed mosquitoes, 122, 33 and 27 genes were found to be significantly up-regulated at 2 hpe, 3 dpe and 14 dpe, respectively. Conversely, the exposure to RVFV resulted in the down-regulation of 217, 27 and 31 genes at 2 hpe, 3 dpe and 14 dpe, respectively. When compared to non- RVFV exposed mosquitoes, most of the DEG (336 genes) were transcriptionally altered at the earliest time point (2 hpe) (Fig 2).

### Functional enrichment of the differentially expressed genes

A functional enrichment analysis of the DEG between the RVFV and control groups was performed using TopGO [52] (S3 and S4 Tables) and Revigo [46] was used to make the charts of the most representative GO categories at each time point tested (S2–S4 Figs). At all-time points evaluated, the GO categories within biological processes related to cellular and metabolic mechanisms were the most affected categories. Within down-regulated DEG, the subcategories related to cellular process, cellular component organization or biogenesis, cellular metabolic process, immune system, multi-organism process or developmental process were more prominent. However, the response to stimulus, cell death, localization, immune system process and developmental process were the most represented GO subcategories within up-regulated DEG.

Out of the 451 DEG, a total of 297 (65.9%) genes were similar to proteins with a functional annotation in the nr NCBI database, as determined by BLASTX; 22.1% were annotated as conserved or hypothetical protein, and 12% did not match with the nr NCBI database.

### Functional prediction of differentially expressed genes related to immunity in response to RVFV exposure

A total of 20 DEG were similar to genes involved in the *Cx*. *quinquefasciatus* mosquito immune system as assessed by the ImmunoDB database. Additionally, another 28 DEG involved in immunity processes were identified using the GO database. Thirteen genes were determined by using both methods. In total, 48 DEG related to antiviral and immune system functions were identified and analyzed in depth (Tables 2, 3 and 4).

**Table 2:** DEG related to the immune infection-defense response at 2 hpe.

*Cx*. *pipiens* SEQ ID	Padj	FC	Sequence length	Blastx results: Best match [Species]*	Conserved domain	E value	IDB.ID accession number	Predicted process/activity	References
Cpip1A029893	4.12E-04	-53.8	581 NT	XP_001842114.1 serine protease [*Culex quinquefasciatus*]**	Lectin_C (pfam00059); CLECT(cd00037)	1.48E-08; 5.21E-10	Cpip: CTL24	Pathogen recognition. C-type lectin (CTL24)	[55–57]
Cpip1A025203	6.06E-04	-39.5	1877 NT	XP_001846442.1 tar RNA binding protein [*Culex quinquefasciatus*]	No pfam; DSRM (cd00048)	3.82E-05	Cpip: SRRP7	miRNA pathway; SRRPs: Small Regulatory RNA Pathway Members (LOQS (R3D1))	[58]
Cpip1A088100	2.34E-03	-23.9	512 NT	XP_001854407.1 gawky *[Culex quinquefasciatus]*	Not found		-	RNAi	[59]
Cpip1A057434	2.00E-91	-19.26	674 NT	XP_001846424.1 proteasome maturation protein [*Culex quinquefasciatus*]	UMP1 (pfam05348)	1.09E-19	**-**	Proteasome assembly;	[60,61]
Cpip1A048037	1.31E-02	-17.6	1416 NT	XP_001842183.1 nuclear pore complex protein Nup155 [*Culex quinquefasciatus*]	nucleoporin N superfamily (pfam08801)	2.51E-38	-	Nuclear transport of transcription factors	[53]
Cpip1A079710	1.59E-05	-14.1	2229 NT	XP_001866714.1 UBX domain-containing protein 8 [*Culex quinquefasciatus*]	UBX (pfam00789);UAS_ETEA (cd02991); Faf1_like2_UBX (cd01774); UBA_FAF2 (cd14414)	0.000121; 7.96E-41; 2.59E-23; 1.08E-10	-	Ubiquitination pathway	[62]
Cpip1A031059	8.51E-03	-12.3	3014 NT	XP_001843599.1 ribonuclease 3 [*Culex quinquefasciatus*]	Ribonuclease_3 (pfam00636); RIBOc (cd00593)	4.41E-16; 5.19E-20	Cpip: SRRP6	miRNA pathway; SRRPs: Small Regulatory RNA Pathway Members (DROSHA)	[63]
Cpip1A009744	1.4E-02	-11.6	574 NT	XP_001842945.1 defensin-A [*Culex quinquefasciatus*]	Defensin_2 (pfam01097)	5 39E-12	Cpip: AMP6	Toll/ IMD Pathways. AMP: Antimicrobial Peptide (DEFA)	[64]
Cpip1A014968	4.31E-02	-11.2	487 NT	AAEL003832-PA [*Aedes aegypti*]	Defensin_2 (pfam01097)	1.12E-06	Cpip: AMP6	Toll/ IMD Pathways. AMP: Antimicrobial Peptide (DEFC)	[64]
Cpip1A005512	3.00E-69	-10.4	1499 NT	XP_021702392.1 leucine-rich repeats and immunoglobulin-like domains protein 3 isoform X2 [*Aedes aegypti*]	LRR_8 (pfam13855); LRR_RI (cd00116)	2.54E-05; 5.67E-04	-	Toll/ IMD Pathways/ Pattern Recognition Receptor	[65,66]
Cpip1A075565	5.55E-06	-8.3	3034 NT	XP_001866729.1 CTP synthase [*Culex quinquefasciatus*]	CTP_synth_N (pfam06418); CTGs (cd03113)	4.91E-85; 2.45E-80	-	Sustainability of activated immune cell proliferation	[54]
Cpip1A027127	2.00E-169	-8.1	1378 NT	XP_001846690.1 tnf receptor associated factor [*Culex quinquefasciatus*]	zf-TRAF (pfam02176)	3.87E-10	-	RNAi; Apoptosis	[67,68]
Cpip1A053625	1.00E-175	-5.0	1238 NT	XP_001842520.1 proteasome subunit alpha type 1 [*Culex quinquefasciatus*]	Proteasome (pfam00227); proteasome_alpha_type_1 (cd03749)	9.77E-37; 8.16e-109	-	Proteasome assembly	[69]
Cpip1A073928	0.00E+00	-4.6	2802 NT	XP_001846422.1 apoptosis inhibitor [*Culex quinquefasciatus*]	API5 (pfam05918)	1.89E-130	-	Apoptosis	[70]
Cpip1A002183	8.04E-03	-4.5	2885 NT	XP_001866729.1 CTP synthase [*Culex quinquefasciatus*]	CTP_synth_N (pfam06418); CTGs (cd03113)	2.54E-85; 1.35E-80	-	Sustainability of activated immune cell proliferation	[54]
Cpip1A091918	5.20E-03	-3.4	2066 NT	XP_001862491.1 piwi [*Culex quinquefasciatus*]	Piwi (pfam02171); Piwi_piwi-like_Euk (cd04658)	2.83E-58; 1.55E-124	Cpip:SRRP33	RNAi; SRRPs: Small Regulatory RNA Pathway Members (PIWI4 transcript 1)	[71,72]
Cpip1A087985	2.99E-02	4.0	2946 NT	KXJ72819.1 hypothetical protein RP20_CCG017164 [*Aedes albopictus*]	PBD (pfam00786); Pkinase (pfam00069); STKc_PAK3 (cd06656)	4.91E-11; 3.92E-47; 7.99E-116	-	Not found immunity function reference. PKC-dependent signaling is a negative regulator of the midgut epithelial barrier	[73]
Cpip1A058921	2.23E-02	8.3	3952 NT	XP_001843328.1 lipin-3 [*Culex quinquefasciatus*]	LNS2 (pfam08235); Lipin_N (pfam04571); Lipin_mid (pfam16876)	3.40E-100; 6.24E-43; 2.32E-20	-	PI3K-Akt-TOR signaling pathway	[74]
Cpip1A000671	2.78E-05	10.3	1398 NT	XP_001844338.1 conserved hypothetical protein [*Culex quinquefasciatus*]	No pfam; P53 (cd08367)	2.70E-21	-	Apoptosis (P53)	[75,76]
Cpip1A039204	1.99E-02	11.1	689 NT	XP_001844330.1 conserved hypothetical protein [*Culex quinquefasciatus*]	E1_DerP2_DerF2 (pfam02221); Npc2_like (cd00916)	3.13E-13; 8.65E-19	Cpip: ML12	Toll/ IMD pathways; MLs: MD2-Like Receptors (ML22)	[77–80]
Cpip1A010903	3.96E-02	15.5	1221 NT	XP_001862507.1 ficolin-2 [*Culex quinquefasciatus*]	Fibrinogen_C (pfam00147); FReD (cd00087)	8.77E-28; 5.02E-43	Cpip: FREP46	Pattern Recognition Receptor	[81]
Cpip1A101513	8.00E-92	18.5	661 NT	XP_001846424.1 proteasome maturation protein [*Culex quinquefasciatus*]	UMP1 (pfam05348)	8.49E-20	-	Proteasome assembly; Apoptosis	[60,61]
Cpip1A065392	1.83E-02	25.4	1135 NT	XP_001843329.1 conserved hypothetical protein [*Culex quinquefasciatus*]	Prefoldin (pfam02996); Prefoldin_alpha (cd00584)	1.21E-06; 6.11E-05	-	RNAi; Virus infection control	[82]
Cpip1A059920	2.64E-02	25.7	1673 NT	XP_001841680.1 chitinase [*Culex quinquefasciatus*]	Glyco_hydro_18 (pfam00704); CBM_14 (pfam01607); GH18_chitolectin_chitotriosidase (cd02872)	2.79E-64; 1.13E-10; 7.50E-127	-		[83]
Cpip1A019311	3.37E-02	27.5	668 NT	XP_001844320.1 MPA2 allergen [*Culex quinquefasciatus*]	E1_DerP2_DerF2 (pfam02221); Npc2_like (cd00916)	2.99E-19; 2.94E-26	Cpip:ML8	Toll/ IMD pathways; MLs: MD2-Like Receptors (ML6)	[77–80]
Cpip1A012593	5.05E-03	27.6	933 NT	XP_001844321.1 conserved hypothetical protein [*Culex quinquefasciatus*]	E1_DerP2_DerF2 (pfam02221); Npc2_like (cd00916)	3.72E-11; 5.23E-09	Cpip:ML16	Toll/ IMD pathways; MLs: MD2-Like Receptors (ML26)	[77–80]
Cpip1A000929	4.04E-02	28.4	3386 NT	XP_001843517.1 fork head [*Culex quinquefasciatus*]	Forkhead (pfam00250); FH (cd00059)	1.34E-29; 3.47E-31	-	TOR (target of rapamycin); Toll and IMD pathways	[84]
Cpip1A054937	3.90E-02	31.3	1894 NT	XP_001865865.1 tetraspanin 97e [*Culex quinquefasciatus*]	Not found			Virus cell-cell spreading. Apoptosis	[85,86]
Cpip1A037043	.,68E-02	37.3	1131 NT	XP_001846625.1 serine protease1/2 [*Culex quinquefasciatus*]	Trypsin (pfam00089); Tryp_SPc (cd00190)	6.46E-46; 2.42E-50	-	Blood digestion	[87]
Cpip1A035296	1.43E-05	37.3	1509 NT	XP_001844339.1 conserved hypothetical protein [*Culex quinquefasciatus*]	No pfam; P53 (cd08367)	3.22E-21	-	Apoptosis (P53)	[75,76]
Cpip1A014760	7.78E-03	48.3	4632 NT	XP_001843517.1 fork head [*Culex quinquefasciatus*]	Forkhead (pfam00250); FH (cd00059)	1.85E-29; 4.78E-31	-	TOR (target of rapamycin); Toll and IMD pathways	[84]
Cpip1A061691	4.61E-06	74.2	939 NT	XP_001846625.1 serine protease1/2 [*Culex quinquefasciatus*]	Trypsin (pfam00089); Tryp_SPc (cd00190)	1.66E-46; 2.04E-51	-	Blood digestion	[87]
Cpip1A083737	2.16E-03	79.7	898 NT	XP_001849502.1 chymotrypsin-2 [*Culex quinquefasciatus*]	Trypsin (pfam00089); Tryp_SPc (cd00190)	5.28E-51; 7.84E-54	-	Blood digestion	[87]
Cpip1A018924	2.19E-03	83.1	448 NT	XP_001862840.1 croquemort [*Culex quinquefasciatus*]	CD36 (pfam01130)	1.05E-28	Cpip:SCR17	Apoptosis/Phagocytosis. SCRs: Scavenger Receptors, Class B (SCRBQ3)	[88]

* First match resulting from the BLASTX search; FC: Fold change; -: no accession number.

**XP_001842114.1 is not a serine protease but a C-type lectin, probable annotation mistake.

**Table 3:** DEG related to the immune infection-defense response at 3 dpe.

*Cx*. *pipiens* SEQ ID	Padj	FC	Sequence length	Blastx results: Best match [Species]*	Conserved domain	E value	IDB.ID accession number	Predicted process/activity	References
Cpip1A095413	4.06E-06	-153.2	1948 NT	XP_001864599.1 ubiquitin-conjugating enzyme [*Culex quinquefasciatus*]	UQ_con (pfam00179); UBCc (cd00195)	4.01E7; 2.22E-09	-	Ubiquitination pathway (E2)	[69,91]
Cpip1A089245	1.15E-02	-81.9	994 NT	AAL78376.1 putative chymotrypsin-like protein [*Culex pipiens pallens*]	Trypsin (pfam00089); Tryp_SPc (cd00190)	8.84E-29; 0E	-	Blood digestion	[87]
Cpip1A065153	4.18E-02	-17.6	1354 NT	AAX59051.1 chymotrypsin-like [*Culex pipiens*]	Trypsin (pfam00089); Tryp_SPc (cd00190)	1.62E-30; 5.74E-29	-	Blood digestion	[87]
Cpip1A069141	3.37E-02	-5.2	967 NT	XP_001862989.1 conserved hypothetical protein [*Culex quinquefasciatus*]	No pfam; SH2_Grb2_like (cd09941)	6.56E-05	-	Insect cytokine signaling in hemocytes. SH2, domain-binding motifs in adaptor protein (P77).	[89,90]
Cpip1A038039	2.75E-02	20.9	655 NT	XP_001844072.1 mlo2 [*Culex quinquefasciatus*]	zf-UBR (pfam02207)	5.63E-05	-	Ubiquitination pathway (E3)	[92,93]
Cpip1A080538	1.72E-02	27.8	839 NT	XP_001844753.1 proteasome activator complex subunit 3 [*Culex quinquefasciatus*]	PA28_beta (pfam02252); PA28_alpha (pfam02251)	1.72E-32; 2.87E-12	-	Proteasome assembly (PA28)	[69,94]

* First match resulting from the BLASTX search; FC: Fold change; -: no accession number.

**Table 4:** DEG related to the immune infection-defense response at 14 dpe.

*Cx*. *pipiens* SEQ ID	Padj	FC	Sequence length	Blastx results: Best match [Species]*	Conserved domain	E value	IDB.ID accession number	Predicted process/activity	References
Cpip1A034914	3.23E-03	-102.5	1416 NT	XP_001848307.1 dihydroxyacetone kinase [*Culex quinquefasciatus*]	Dak1 (pfam02733)	5.72E-23	-	Virus sensor inhibition	[96]
Cpip1A097556	4.71E-02	-29.5	3834 NT	XP_019527564.1 PREDICTED: heat shock 70 kDa protein 4-like isoform X2 [*Aedes albopictus*]	HSP70 (pfam00012); NAD-GH (pfam10712); HSPA4_like_NDB (cd10228)	2.33E-100; 2.58E-08; 4.11E-149	-	RNAi; Virus infection control	[95,97,98]
Cpip1A099497	2.24E-02	-29.0	3556 NT	XP_001851487.1 lamin [*Culex quinquefasciatus*]	Treacle (pfam03546); Filament (pfam00038); LTD (pfam00932); Treacle (pfam03546); BAR_SNX (cd07596)	1.65E-39; 5.15E-04; 5.02E-03; 1.13E-06	-	Melanization	[99]
Cpip1A075447	1.01E-02	-20.5	4348 NT	XP_001652595.1 cullin-3 isoform X2 [*Aedes aegypti*]	Cullin (pfam00888); Cullin_Nedd8 (pfam10557)	1.21E-168; 2.36E-17	-	Ubiquitination pathway (E3)	[93,100,101]
Cpip1A046835	3.02E-03	-12.2	3441 NT	XP_001851487.1 lamin [*Culex quinquefasciatus*]	Treacle (pfam03546); Filament (pfam00038); BAR_SNX (cd07596); LTD (pfam00932)	4.84E-03; 1.58E-39; 1.09E-06; 4.97E-04	-	Melanization	[99]
Cpip1A086629	2.01E-03	-10.7	301 NT	XP_001848473.1 salivary C-type lectin [*Culex quinquefasciatus*]	CLECT (cd00037)	8.03E-10	Cpip: CTL27	Pathogen recognition. C-Type Lectins (CTL27)	[55–57]
Cpip1A019555	1.05E-02	23.3	1224 NT	XP_001847908.1 microfibril-associated glycoprotein 4 [*Culex quinquefasciatus*]	Fibrinogen_C (pfam00147); FReD (cd00087)	4.42E-54; 1.33E-71	Cpip: FREP18	FREPs: Fibrinogen-Related Proteins (FREP18). Pattern Recognition Receptor	[81]
Cpip1A005797	4.40E-03	78.9	1051 NT	XP_019551901.1 PREDICTED: nuclear pore complex protein Nup88-like [*Aedes albopictus*]	Nup88 (pfam10168)	2.94E-52	-	Nuclear transport of transcription factors; Toll pathway	[102]

* First match resulting from the BLASTX search; FC: Fold change; -: no accession number.

#### DEG related to the immune response at 2 hpe to RVFV

A total of 16 DEG related to immunity were down-regulated at 2 hpe (Table 2). These included genes encoding two antimicrobial peptides (AMP) (Cpip1A009744 and Cpip1A014968), one C-type lectin (CTL) (Cpip1A029893), one leucine-rich repeats (LRR) protein (Cpip1A005512), three small regulatory RNA pathway (SRRPs) members (Cpip1A091918, Cpip1A031059 and Cpip1A025203), two proteins involved in RNAi pathway (Cpip1A088100 and Cpip1A027127), two proteins implicated in the proteasome assembly pathway (Cpip1A057434 and Cpip1A053625), one protein related to the ubiquitination pathway (Cpip1A079710), and one protein involved in apoptotic process (Cpip1A073928). Finally, one nuclear pore complex protein Nup155 (Cpip1A048037) [53], and two CTP synthases (Cpip1A002183 and Cpip1A075565) [54], similar to genes putatively related to antiviral/immune response processes, and with no previous description in insects were identified.

A total of 18 DEG related to immunity were up-regulated at 2 hpe. Four of their putative protein products had homology with three members of myeloid differentiation 2-related lipid recognition (MLs) receptors (MD2-like) (Cpip1A012593, Cpip1A019311 and Cpip1A039204) and one member of fibrinogen-related proteins (FREPs) (Cpip1A010903). Whereas MD2-like proteins are associated with the Toll pathway, FREPs, which are involved in pathogen recognition, have not been linked to any specific immune pathway [22]. Moreover, DEG coding for two fork head (FKH) transcription factors involved in AMP regulation via the TOR pathway (Cpip1A000929 and Cpip1A014760), one lipin-3 (Cpip1A058921) involved in PI3K-Akt-TOR signaling pathway, one protein related to proteasome assembly (Cpip1A101513) and three proteins involved in apoptotic process (Cpip1A000671, Cpip1A035296, and Cpip1A018924) were also up-regulated. The gene Cpip1A018924 had a best match with *Cx*. *quinquefasciatus* Croquemort (SCRBQ3), a scavenger receptor B member of a multigene family harboring the CD36 domain. When screening the whole *Cx*. *pipiens* transcriptome, six other genes homologous to Cpip1A018924 were found; all coded for the CD36 domain. Three additional up-regulated DEG were identified as trypsins (Cpip1A06169, Cpip1A037043 and Cpip1A083737). Finally, the putative products of Cpip1A054937, Cpip1A065392, Cpip1A059920 and Cpip1A087985, which are homologous to proteins involved in diverse responses to viral and antimicrobial infections were also up-regulated.

#### DEG related to the immune response at 3 dpe to RVFV

A total of four DEG involved in insect immunity were down-regulated at 3 dpe (Table 3): one encoding for a protein related to the ubiquitination pathway (Cpip1A095413), one related to alternative antiviral response, with a SH2_Grb2_like protein domain (Cpip1A069141) and two DEG encoding for chymotrypsin-like proteins (Cpip1A089245 and Cpip1A065153). The SH2 domains are implicated in signal transduction and the Grb2 is supposed to play a role in apoptosis [89,90]. In the opposite direction, two DEG implicated in ubiquitination and proteasome assembly pathways were upregulated at 3 dpe (Cpip1A038039 and Cpip1A080538, respectively).

#### DEG related to the immune response at 14 dpe to RVFV

A total of six DEG related to immunity were down-regulated at 14 dpe (Table 4). These DEG included genes encoding one CTL protein (Cpip1A086629), one protein involved in the ubiquitination pathway (Cpip1A075447), two proteins related to melanization (Cpip1A099497 and Cpip1A046835), one heat shock protein 70 (Hsp70) (Cpip1A097556), which has been described previously in mosquito immune response [95], and finally, one protein (Cpip1A034914) that inhibits a virus sensor described in humans [96]. In the other direction, two DEG related to immunity response were up-regulated at 14 dpe. These genes encode one FREP member (Cpip1A019555) and the nuclear pore complex protein Nup88-like (Cpip1A005797) related to antiviral/immune response process in insects.

### Evolution of the immune response upon RVFV exposure in *Culex pipiens* mosquitoes

**Fig 3:**
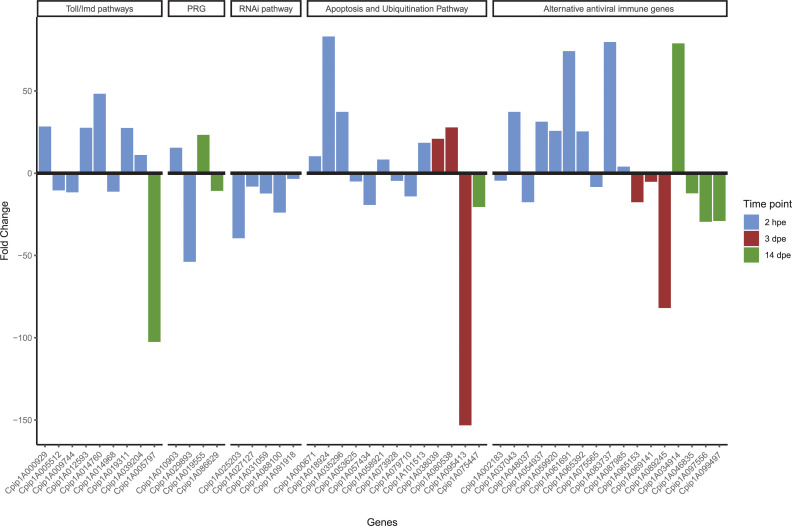
Evolution of the immune infection-response upon RVFV exposure in *Cx*. *pipiens* mosquitoes. The immune DEG altered at 2 hpe, 3 and 14 dpe are represented in blue, red and green colors, respectively. PRG: Pathogen recognition genes.

All of the above detailed DEG during RVFV-exposed in mosquitoes were involved in the conventional immune Toll/IMD pathways, RNAi mechanism, the ubiquitination pathway, apoptosis and other inducible antiviral immune genes (Fig 3). Most of the transcriptional changes related to immunity occurred at the early RVFV-infection stage (2 hpe) in all the defense mechanisms. It is worth mentioning that all DEG related to the RNAi system were down-regulated at this time point (Fig 3).

## Discussion

The full genome sequence of *Cx*. *pipiens* is not available, therefore, in the present study we constructed a *de novo* transcriptome using adult *Cx*. *pipiens* females fed on RVFV infectious or non-infectious blood allowing us to perform an accurate comparative transcriptome analysis between RVFV exposed and unexposed female mosquitoes.

Most of the transcriptomic alterations were found within 2 hpe, at an early stage of infection. Most of the DEG identified at this stage were enriched in GO categories corresponding to metabolic and cellular processes most likely at a time when the mosquito midgut is primed with RVFV. These results are in line with previous findings obtained with ZIKV-infected *Ae*. *aegypti* mosquitoes [103], midguts of DENV-infected *Ae*. *aegypti*, and midguts of CHIKV- infected *Ae*. *albopictus* [104,105], where most of the altered genes were detected at the beginning of the infection. Among all DEG, we focused specifically on genes putatively involved in the mosquito immune system since antiviral mechanisms very often do not entirely clear the virus but allow the establishment of a persistent viral infection in vectors. In the present study, 48 DEG belonging to several infection defense gene families have been identified upon RVFV exposure. These genes are related to Toll, IMD and RNAi pathways as well as infection-responsive pathogen recognition genes (PRG), apoptotic processes, and other alternative inducible antiviral immune genes.

One of the limitations of our study has been to analyze the entire mosquito instead of specific tissues. This could explain why some DEG related to the immune response have been regulated in opposite directions. Moreover, since we pooled mosquitoes without knowing the infection status of each individual, thus introducing heterogeneity and reducing the power to detect DEG, other genes could have been altered after RVFV exposure but were not detected in our study, essentially at 3 and 14 dpe.

### Early depletion of the RNAi pathway by RVFV exposure

The RNAi pathway seems suppressed at the onset of the RVFV infection in *Cx*. *pipiens* since crucial SRRP members such as Piwi4, Loquacious (Loqs or R3D1), ribonuclease 3 (Drosha), tnf receptor associated factor (zf-TRAF) and Gawky were down-regulated at 2 hpe.

The Piwi4 protein, which belongs to the PIWI family, interacts with key components of the siRNA (Ago2 and Dicer-2 proteins) and piRNA (Ago3, Piwi5 and Piwi6 proteins) pathways, although it has been proposed that its antiviral role is independent of either pathway [72]. Piwi4 has been involved in the control of Semliki Forest virus and Bunyamwera virus in *Ae*. *aegypti* and *Ae*. *albopictus*-infected mosquito cells, respectively [106,107]. Moreover, Dietrich *et al*. (2017) also demonstrated the antiviral activity of Piwi4 in Aag2 cells infected with RVFV since its silencing led to an increase of viral replication [71]. According to these studies, the depletion of Piwi4 mRNA could indicate that RVFV suppresses the piRNA pathway allowing its replication in *Cx*. *pipiens* mosquitoes at an early stage of infection. Additionally, the mRNA coding for the Loqs protein, was also down-regulated. Haac *et al*. (2015) proposed that in dipterans, including mosquitoes, Loqs-PA and Loqs-PB isoforms are involved in the control of the miRNA pathway [58]. Therefore, RVFV may also alter the miRNA pathway in *Cx*. *pipiens* via Loqs downregulation early after RVFV exposure.

Other components of the miRNA and RNAi pathways were down-regulated: the *Drosha* gene, which codes for an enzyme that generate small RNAs in cooperation with Dicer to control viral infections [63], and Gawky, an Argonaute-associating protein whose role in the miRNA pathway repression is still not clear [59]. Finally, ending the list of down-regulated genes at 2 hpe, TRAFs has been implicated in the activation of the *Culex* Vago protein, which is a secreted peptide with antiviral functions [108] in response to WNV in infected mosquito cells [68].

In summary, the depletion of Piwi4, Loqs, Drosha, Gawky and TRAF, all involved in several RNAi pathways, would suggest a severe alteration of this pathway at the onset of the RVFV infection to allow viral replication in the intestinal barrier. On the other hand, transcription of genes involved in the RNAi pathway were not affected in the later sampled time points (at 3 and 14 dpe), suggesting that, once the initial RVFV infection is established, steady state levels of these genes allows proper dissemination of the virus without causing lethal tissue damages in *Cx*. *pipiens*. In addition to the detection and quantification of siRNAs and piRNAs, further functional studies will be required to better understand the role of the RNAi pathway in RVFV-infected *Cx*. *pipiens* mosquitoes to evidence the presence or absence of a RVFV potential inhibitor of this pathway. Moreover, studies on how RVFV NSs protein can be considered as a Virus Suppressors of RNAi should be also investigated as for other bunyaviruses [109].

### RVFV exposure is associated with changes in *Cx*. *pipiens* immune responses, mainly in the expression of Toll and IMD pathways related genes

Genes belonging to the Toll and IMD pathways coding for defensin-A (DEFA), defensin-C (DEFC) and ML proteins (ML6, ML22, ML26) were significantly positively or negatively altered throughout the course of RVFV infection in *Cx*. *pipiens* mosquitoes. Conversely, none of the DEG were related to the JAK/STAT pathway, contrary to what has been reported in several studies performed in *Ae*. *aegypti* with other arboviruses (ZIKV and DENV) [22,110] or *Cx*. *quinquefasciatus* with WNV [111]. However, our results agree with two previous studies performed in *Ae*. *albopictus* infected with CHIKV [105] and in *Ae*. *aegypti* infected with ZIKV [103] where the JAK/STAT pathway was not involved in virus-mosquito interactions. These apparent contradictory data suggest that the implication of the JAK/STAT pathway in arbovirus defense depends on the pairing combination of mosquito and arbovirus and is influenced by the viral genotype—host genotype interaction, as previously was proposed for different RVFV strains by Pinkham *et al*. (2017) [112].

The AMPs are induced following activation of Toll, IMD and JAK/STAT pathways. They are potent immune effectors whose ultimate function is to clear pathogens [113]. Their mode of action is better understood for bacteria and fungi, but how they interact with viruses remains unclear. In the present study, two AMPs, defensin-A (DEFA) and defensin-C (DEFC), were under-expressed in RVFV exposed females at the beginning of the infection (at 2 hpe). Defensins are mainly known to disrupt the membrane permeability of Gram- positive bacteria A previous study performed in *Ae*. *aegypti* infected with ZIKV or CHIKV showed that DEFA and DEFC were over-expressed at 3 hpe to both viruses. However, at 10 dpe, in comparison with uninfected controls, DEFA and DEFC were significantly down-regulated in CHIKV and up-regulated in ZIKV infected females, respectively [64]. Regulation of defensins seems to vary depending on the virus ingested and the progression of the infection. RVFV probably modifies the expression of both defensins in order to allow initiation of the infection in *Cx*. *pipiens*, but returns to steady state levels when the virus crosses the midgut barrier. According to results obtained in *Drosophila*, up-regulation of the transcription factor FKH, which acts downstream of TOR signaling, inhibits production of defensins while increasing other AMPs mainly diptericin and metchnikowin [84]. However, no other AMP genes were affected in RVFV-exposed females. Furthermore, members of the ML family (ML22, ML6 and ML26 genes) were up-regulated in RVFV-exposed mosquitoes at 2 hpe. ML genes, which encode lipid-binding proteins, have been implicated in host-pathogen interactions. Currently, the role of the ML protein family in mosquito immunity is still not well understood [79,80]. For instance, Jupatanakul *et al*. (2014) showed that when the ML33 gene is silenced in DENV infected *Ae*. *aegypti* females, virus titers in midguts are reduced, indicating that the ML33 gene facilitates viral infection in the mosquito midgut [78]. Our results may also suggest that ML22, ML6 and ML26 could influence the outcome of a RVFV infection in *Cx*. *pipiens* females since they were up-regulated at an early stage of infection. Also, a gene coding for a LRR-containing domain protein was down-regulated in RVFV infected females at 2 hpe. A recent study by Zhao *et al*. (2019) has shown that the expression of *AaeLRIM1* and *AaeAPL1* (coding for proteins containing LRR) were also down-regulated in ZIKV infected *Ae*. *aegypti* at 7 dpi [65], which is in agreement with our results. However, it is unknown how these proteins interact with arboviruses [65] therefore functional studies are required to better understand the role of LRR immune proteins against arboviruses.

Additionally, the nuclear pore complex protein Nup88 seems to promote nuclear retention of immune regulators, such as the transcription factors Dif and Dorsal, contributing to an efficient control of the immune response when the Toll pathway is engaged upon microbial infection [102]. However, at this time point (14 dpe) no other members of the Toll pathway were altered in *Cx*. *pipiens* RVFV-infected females.

### RVFV alters *Cx*. *pipiens* infection-responsive pathogen recognition genes

The FREPs and CTLs have not been directly classified within any of the conventional immune pathways but are described as infection-responsive pathogen recognition proteins and as such play a role in mosquito immunity response [22,81]. A study performed in *Ae*. *aegypti* mosquitoes infected with ZIKV showed that the FREP37 gene was down-regulated at 7 dpe [22]. Our study indicates over-expression of FREP46 and FREP18 genes at 2 hpe and 14 dpe, respectively. However, we cannot assess whether the regulation of FREP genes is affected one week after infection since no intermediary time point was performed between 3 dpe and 14 dpe. In addition, the CTL24 and CTL27 transcripts were depleted in RVFV-infected females at 2 hpe and at 14 dpe. The CTLs play an essential role as pattern recognition receptors to mediate immune responses, such as phagocytosis and melanization [55,114]. A previous study demonstrated that *Ae*. *aegypti* (*mosGCTL-1*) and *Cx*. *quinquefasciatus* (*Culex mosGCTL-1*) CTLs were overexpressed and facilitated WNV infection in adult mosquitoes at six days post-inoculation (dpi) [56]. The same results were obtained on DENV infected *Ae*. *aegypti* mosquitoes [57]. Accordingly, our results suggest that CTL24 and CTL27 may mitigate the RVFV replication in *Cx*. *pipiens* mosquitoes at different stages of the infection.

### RVFV infection alters expression of genes related to the ubiquitination pathway and apoptosis

The UPP, which recognizes and degrades polyubiquitylated proteins modified via ubiquitination, contributes to multiple biological processes including apoptosis [69], an important immune mechanism that modulates viral infections in vertebrates and invertebrates [115]. Furthermore, it was observed that *in vivo*, silencing of some proteasome catalytic β sub-units reduced the number of DENV infected mosquitoes, and upregulation of UPP-related genes were required in *Ae*. *aegypti* midguts for the production of infectious DENV [61]. Therefore, it appears that a functional proteasome is required to generate viral particles [61].

In the present study, deprivation of the proteasome subunit alpha and reduced dimerization of precursor complexes containing β subunits, mediated by ubiquitin-mediated proteolysis (UMP1a) [60], combined with a deficiency of ubiquitin-activating enzymes (UBX/UBA or E1), which initiate the ubiquitination pathway cascade [62], would alter the formation and function of proteasomes in *Cx*. *pipiens* infected by RVFV. However, the up-regulation of a gene coding for UMP1b could counterbalance the effect of UMP1a. At the present state of knowledge, it is difficult to assess if UMP1a and UMP1b are two isoforms of the same gene or the products of duplicated genes. The fact that they are oppositely regulated argues for the existence in *Cx*. *pipiens* of two distinct UMP1 genes. The involvement of E1 enzymes in infectious processes has also been previously described for DENV infected *Ae*. *aegypti* midguts, but in this case, up-regulation of these genes probably exerted a beneficial role for the development of the virus [61].

At 3 dpe one DEG coding for one ubiquitin-conjugating enzyme (UBC) or E2, belonging to the ubiquitination pathway in *Drosophila* [69,91], was depleted. At this time point, the ubiquitin ligase containing the UBR box (zf-UBR) or E3 [93,101], and the proteasome activator complex subunit 3 (PA28) were enriched in RVFV infected females. As far as we know, neither of these two proteins have thus far been implicated in arbovirus infectious processes in dipterans. However, in mammals, PA28, a regulatory complex protein induced by IFN-γ [94], has been shown to interfere with the replication of coxsackievirus B3 in human and murine cells by suppressing viral replication [94]. Overall, results obtained in this study suggest that the UPP and the proteasome could affect the developmental cycle of RVFV. Six DEG would inhibit virus replication while one up-regulated DEG (the ubiquitin ligase of the E3 family) could enhance it. However, experimental proof is needed to ascertain the role of the above-mentioned genes during RVFV infection.

As mentioned above, the UPP and proteasome are tightly related to the apoptotic process. The role of apoptosis in arboviral infections is still subject to debate. For example, apoptosis occurring in the midgut epithelium and salivary glands of *Cx*. *pipiens* and *Cx*. *quinquefasciatus* respectively, induced resistance to WNV infection [25] and reduced transmissibility of WNV [116] while silencing the apoptosis inhibitor IAP1 in *Ae*. *aegypti* resulted in an increment of SINV infection [35].

In the present study, we detected a certain number of genes related to apoptosis up or down-regulated and exerting opposite effects. However, we do not know if apoptosis takes place; this will require specific experiments in different organs or tissues, such as midgut, fat body or salivary glands of RVFV infected females. However, the fact that most of the DEG implicated here in apoptosis or UPP were found at an early stage of infection would suggest, that if apoptosis does occur, then it will take place in *Cx*. *pipiens* midguts. It is important to highlight the lack of understanding about the effect of apoptosis during arboviral infections. Further experiments will be necessary to clarify how apoptosis contributes to RVFV infection in *Cx*. *pipiens* mosquitoes.

### Alternative inducible antiviral immune genes

In the present study, some DEG with no conventional immune pathway attribution but which are involved in immune responses against pathogens were identified. Trypsins, Hsp70 and lamin belong in this last category. The trypsins, which are blood-digestive enzymes, were overexpressed within the first hours of infection and were down-regulated at 3 dpe. Our results suggest that blood digestion and probably RVFV proteolytic processing via midgut trypsins may facilitate RVFV infection as previously reported by Molina-Cruz *et al*. (2005) in DENV-2 infected *Ae*. *aegypti* [87]. At a stage when the RVFV infection is already established in *Cx*. *pipiens* mosquitoes (at 14 dpe), a gene coding for Hsp70 was under-expressed. The chaperone protein Hsp70 facilitates infection of multiple viruses in mammalian cells, such as DENV [97], Japanese encephalitis virus [98] or ZIKV [95]. Moreover, Pujhari *et al*. (2018) have recently demonstrated that the Hsp70 protein has an important role in the cell cycle during infection, as well as viral entry, replication and egress. Additionally, they showed that the inhibition of Hsp70 led to decreased ZIKV titer in infected Huh7.5 human cells [95]. By analogy, our results indicate that lower levels of Hsp70 might limit but not abolish RVFV production to increase mosquito viability. Finally, two *lamin* genes were under-expressed at 14 dpe in RVFV infected mosquitoes. The *lamin* genes have been beforehand identified as involved in melanization in *Drosophila* [99], but their potential antimicrobial activity in mosquitoes and arboviral infections need to be confirmed.

Transcriptomic studies are crucial for detecting genes implicated in mosquito vector competence for arboviruses, since new control strategies can be drawn. To date, no comprehensive transcriptomic analysis had been performed in mosquitoes with this particular zoonotic virus. Other mosquito species shown to be RVFV vectors combined with other RVFV strains from different genetic and geographic lineages and broader range of time intervals should also be analyzed for transcriptomic analysis to enlarge the knowledge on RVFV-mosquito vector interaction. The present study significantly expands our understanding of *Cx*. *pipiens* and RVFV interactions, proposing possible roles of conventional immune pathways (Toll, IMD, RNAi, UPP and apoptosis) in the control or exacerbation of the infection. Moreover, other inducible antiviral genes were modulated upon RVFV exposure in *Cx*. *pipiens* females. At an early stage of infection, some crucial defense effectors are inhibited, providing an opportunity for RVFV to disseminate. Thus, the present work provides a number of target genes and hypotheses on which to base future functional studies of the mechanisms inducing viral replication and resistance to RVFV infection.

## Supporting information

S1 Fig Representation of the PCA results. (EPS)

S2 Fig Representation of Gene Ontology categories assigned to DEG at 2 hpe during RVFV infection in *Cx*. *pipiens*. A) Main GO categories of down-regulated DEG at 2 hpe. The legend shows the GO categories (GO1 to GO5). The most represented were cellular process and cellular component organization or biogenesis. B) Principal GO categories of up-regulated DEG at 2 hpe. In the legend the GO categories (GO1 to GO7) are shown. The most represented corresponded to cellular process, response to stimulus, metabolic process and cell death.
(TIF)

S3 Fig Representation of Gene Ontology categories assigned to DEG at 3 dpe during RVFV infection in *Cx*. *pipiens*. C) Main GO categories of down-regulated DEG at 3 dpe. The legend shows the GO categories (GO1 to GO4). The most represented corresponded to multi-organism process, cellular response to stimulus, cellular metabolic process and developmental process. D) Principal GO categories of up-regulated DEG at 3 hpe. In the legend the GO categories (GO1 to GO3) are shown. The most represented categories were cellular metabolic process, localization and multicellular organismal process.
(TIF)

S4 Fig Representation of Gene Ontology categories assigned to DEG at 14 dpe during RVFV infection in *Cx*. *pipiens*. E) Principal GO categories of down-regulated DEG at 14 dpe. The legend shows the GO categories (GO1 to GO4). The most represented corresponded to immune system process, cellular component organization or biogenesis, development process and localization. F) Main GO categories of the up-regulated DEG at 14 dpe. In the legend the GO categories (GO1 to GO6) are shown. The most represented were cellular component organization or biogenesis, metabolic process, cellular metabolic process, immune system process and developmental process.
(TIF)

S1 Table Results of the QC mapping metrics. (XLSX)

S2 Table DEG during RVFV infection in *Cx*. *pipiens* mosquitoes. The transcripts differentially expressed at 2 hpe, 3 dpe and 14 dpe were listed with corresponding best matches in the protein nr database at NCBI. Sequences of the transcripts were subjected to BLASTX with a cut-off of P value < 0.0001. Open Reading Frame (ORF) annotations (*) were obtained by combining a search performed with the Pfam, Conserved Domain and Swiss-prot databases. The DEG highlighted in green colour corresponds to the immune related DEG identified in the present study.FC = fold change; nr = non-redundant;— = no significant.
(XLSX)

S3 Table Down-regulated DEG at 2 hpe and at 3 and 14 dpe with their respective GO categories in RVFV infected *Cx*. *pipiens*. DEG were assigned to GO functional categories for biological process **using TopGO software with** Fisher’s exact test**. The cut-off for ‘classicFisher’ was P-value <0.05.**
(XLSX)

S4 Table Up-regulated DEG at 2 hpe and at 3 and 14 dpe with their respective GO categories in RVFV infected *Cx*. *pipiens*. DEG were assigned to GO functional categories for biological process **using TopGO software with** Fisher’s exact test**. The cut-off for ‘classicFisher’ was P-value <0.05**
(XLSX)
