# Supplementary figures and images for: Alteration in the Culex pipiens transcriptome reveals diverse mechanisms of the mosquito immune system implicated upon Rift Valley fever phlebovirus exposure
Source: PLoS Negl Trop Dis. 2020 Dec 10;14(12):e0008870. doi: 10.1371/journal.pntd.0008870 (PMC7755283; doi:10.1371/journal.pntd.0008870)

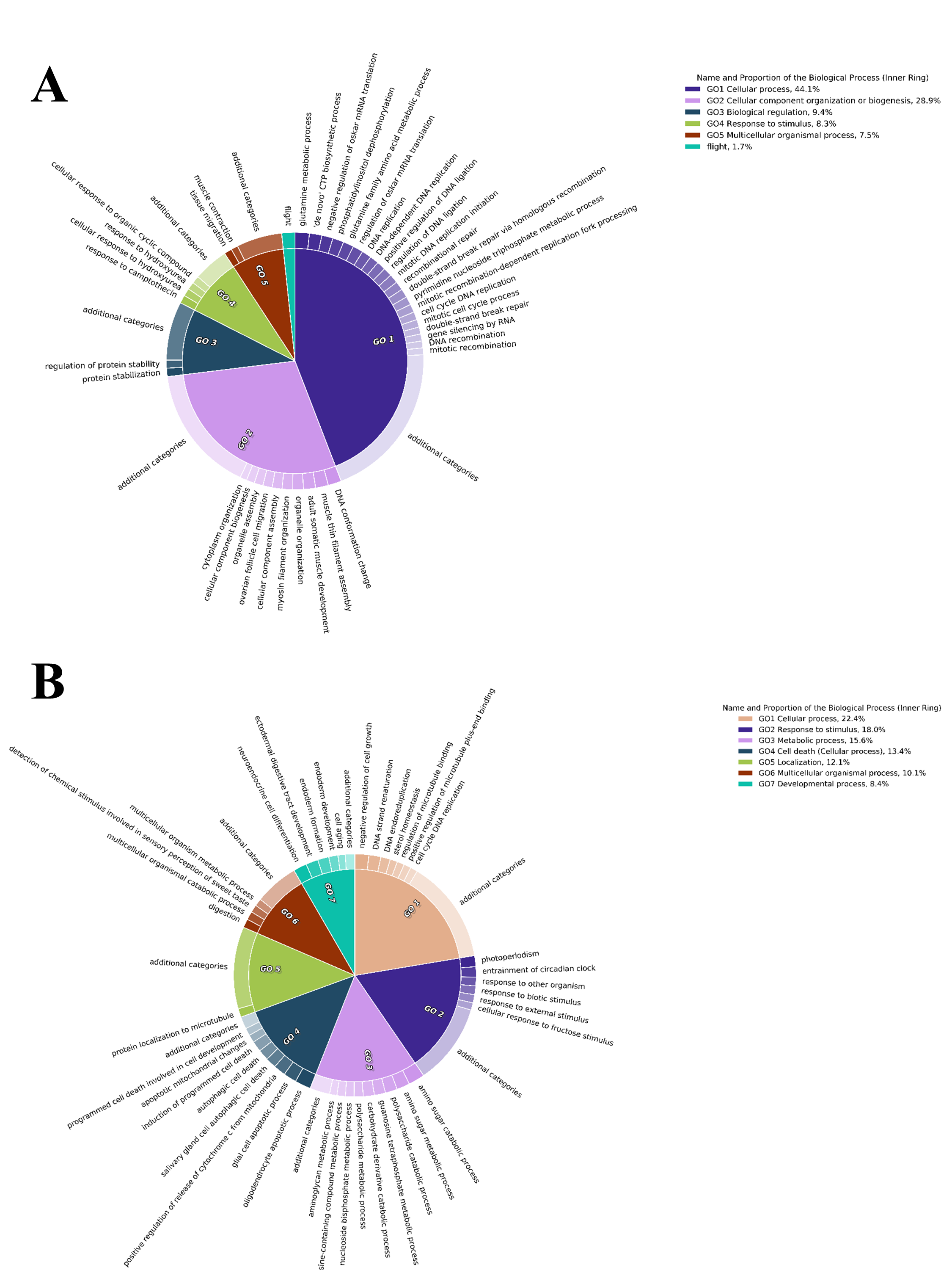

Supplement: S2 Fig — A) Main GO categories of down-regulated DEG at 2 hpe. The legend shows the GO categories (GO1 to GO5). The most represented were cellular process and cellular component organization or biogenesis. B) Principal GO categories of up-regulated DEG at 2 hpe. In the legend the GO categories (GO1 to GO7) are shown. The most represented corresponded to cellular process, response to stimulus, metabolic process and cell death. (TIF) [file pntd.0008870.s002.tif]

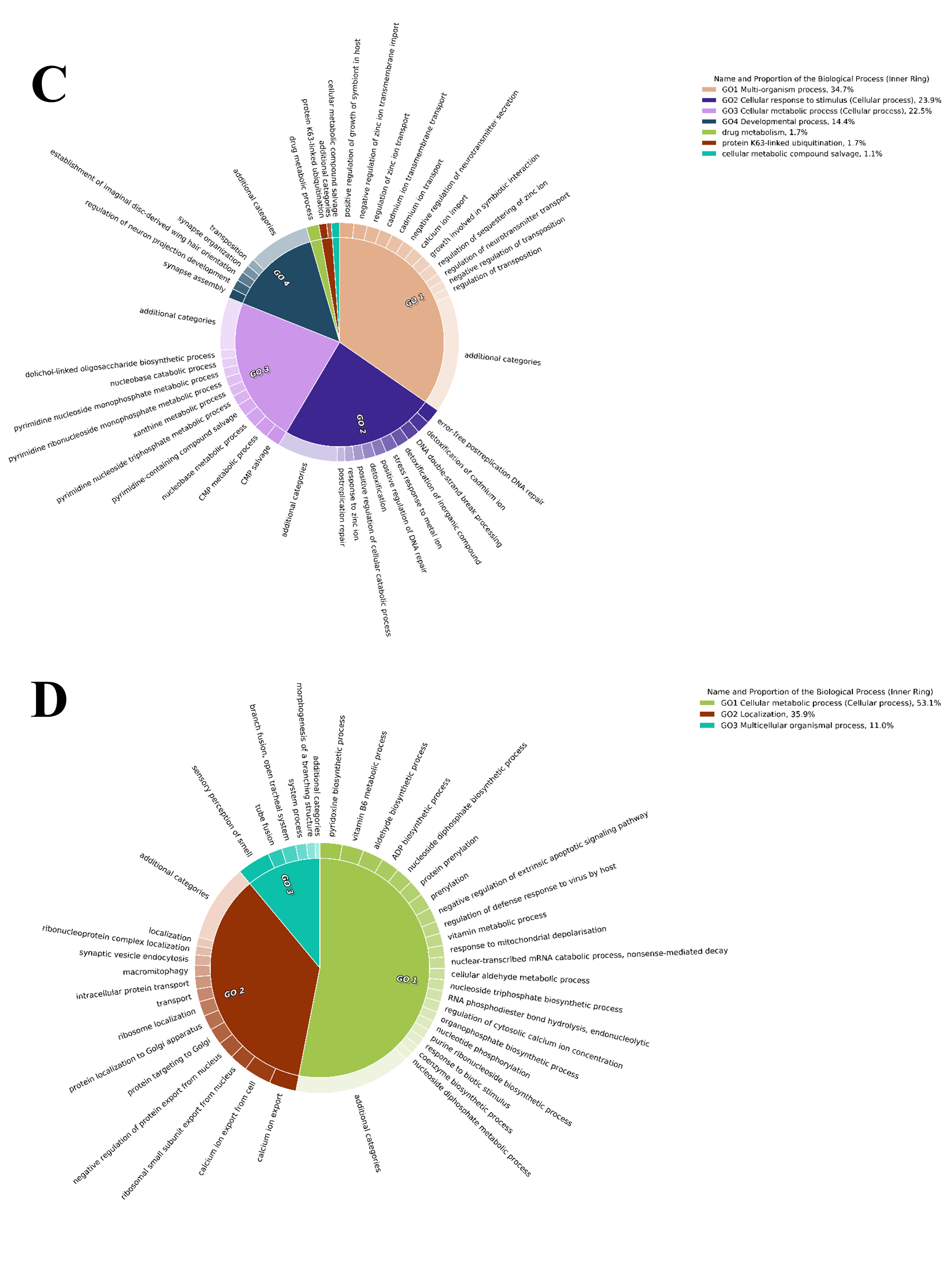

Supplement: S3 Fig — C) Main GO categories of down-regulated DEG at 3 dpe. The legend shows the GO categories (GO1 to GO4). The most represented corresponded to multi-organism process, cellular response to stimulus, cellular metabolic process and developmental process. D) Principal GO categories of up-regulated DEG at 3 hpe. In the legend the GO categories (GO1 to GO3) are shown. The most represented categories were cellular metabolic process, localization and multicellular organismal process. (TIF) [file pntd.0008870.s003.tif]

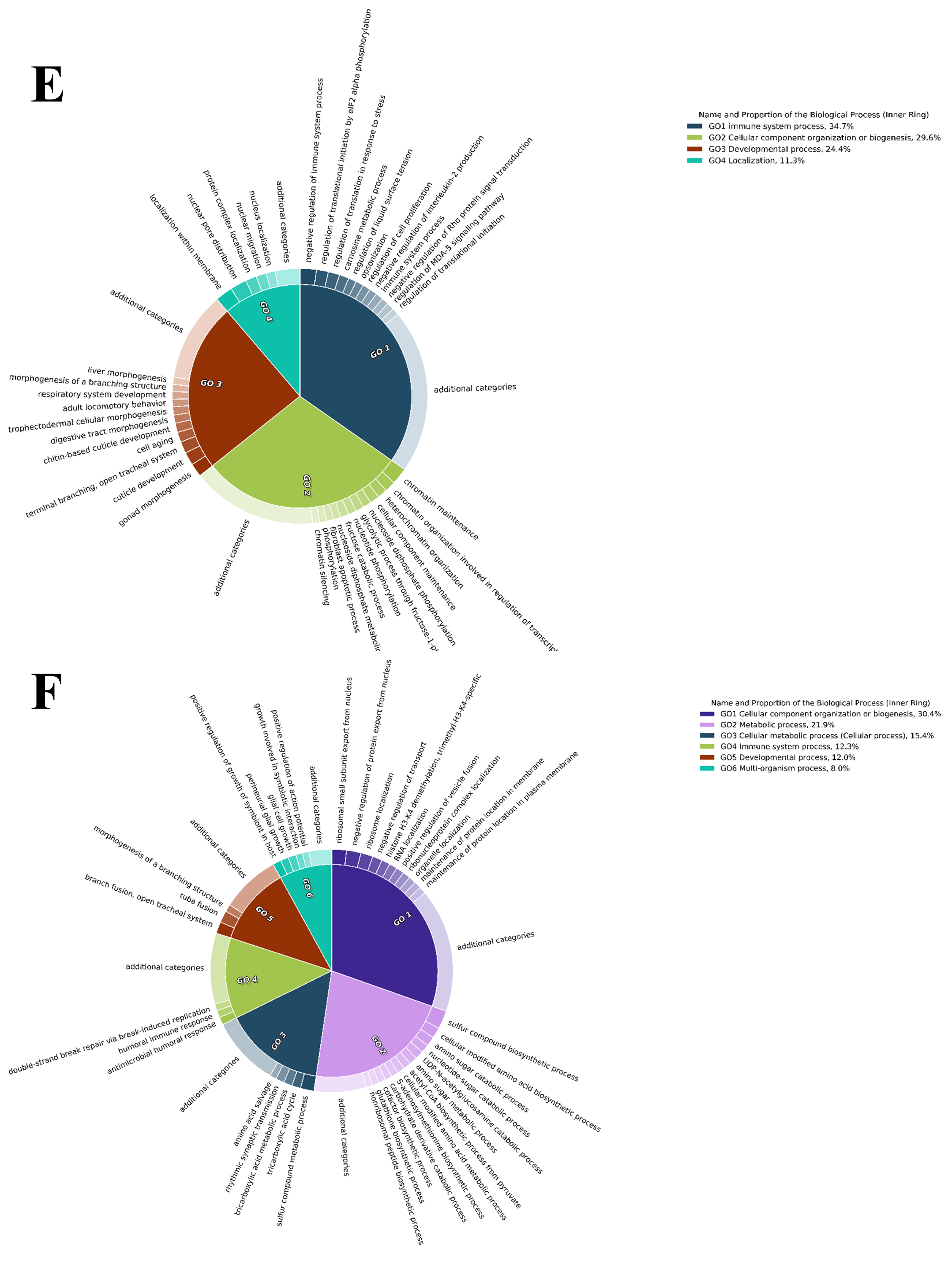

Supplement: S4 Fig — E) Principal GO categories of down-regulated DEG at 14 dpe. The legend shows the GO categories (GO1 to GO4). The most represented corresponded to immune system process, cellular component organization or biogenesis, development process and localization. F) Main GO categories of the up-regulated DEG at 14 dpe. In the legend the GO categories (GO1 to GO6) are shown. The most represented were cellular component organization or biogenesis, metabolic process, cellular metabolic process, immune system process and developmental process. (TIF) [file pntd.0008870.s004.tif]
